# Supplementary material for: Active gaze behavior organizes V1 activity in freely-moving marmosets
Source: bioRxiv. 2026 Feb 7:2026.02.05.704079. Preprint. [Version 1] doi: 10.64898/2026.02.05.704079 (PMC12889743; doi:10.64898/2026.02.05.704079)
Supplement: 1 [file NIHPP2026.02.05.704079V1-supplement-1.pdf]

bioRxiv preprint doi: https://doi.org/10.64898/2026.02.05.704079; this version posted February 7, 2026. The copyright holder for this preprint (which was not certified by peer review) is the author/funder, who has granted bioRxiv a license to display the preprint in perpetuity. It is made available under aCC-BY 4.0 International license.

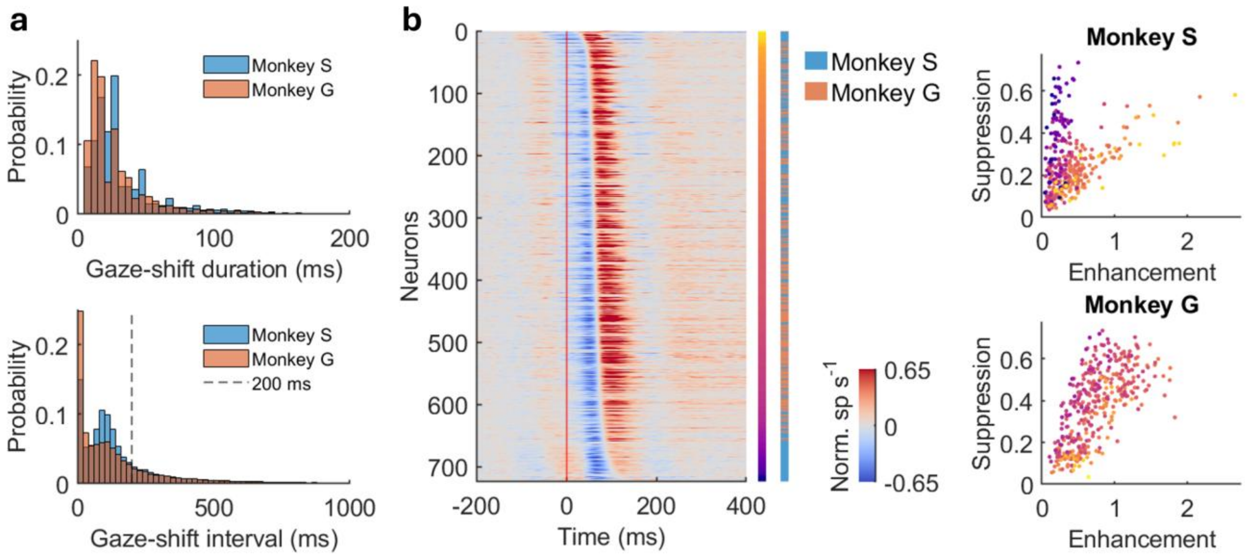

Figure S1. Statistics of gaze-shift behavior and gaze-shift aligned neural responses across two monkeys.

a) Histogram of gaze-shift duration (top) and interval (bottom) for the two monkeys. A large amount gaze-shift intervals fall within 200 ms, indicating frequent gaze shifts during active exploration of the 3D environment.

b) Left: normalized PSTHs aligned with gaze-shift onset (same as Fig. 1d) with identity of monkeys shown by the vertical bar. Right: suppression amplitude versus enhancement amplitude (same as Fig. 1g) for each monkey. Each dot represents a neuron with color indicating latency as in b. The conclusion of larger suppression/enhancement ratio for later neurons than earlier neurons hold in each monkey.

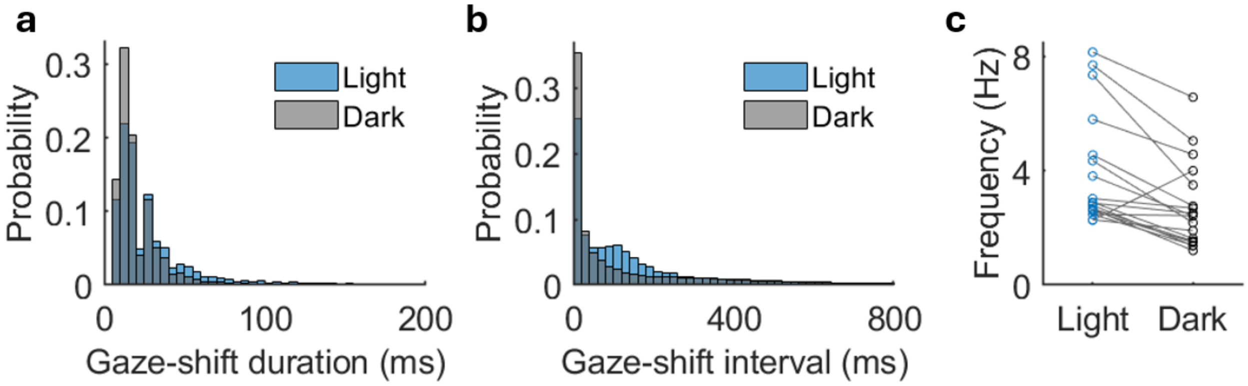

Figure S2. Statistics of gaze-shift behavior in light versus dark.

a) Histogram of gaze-shift duration in the light and dark.

b) Histogram of gaze-shift interval in the light and dark.

c) Average frequency of gaze shifts in the light and dark for each session. Gaze shifts in the dark are generally less frequent than in the light but remain at a decent level.

bioRxiv preprint doi: https://doi.org/10.64898/2026.02.05.704079; this version posted February 7, 2026. The copyright holder for this preprint (which was not certified by peer review) is the author/funder, who has granted bioRxiv a license to display the preprint in perpetuity. It is made available under aCC-BY 4.0 International license.

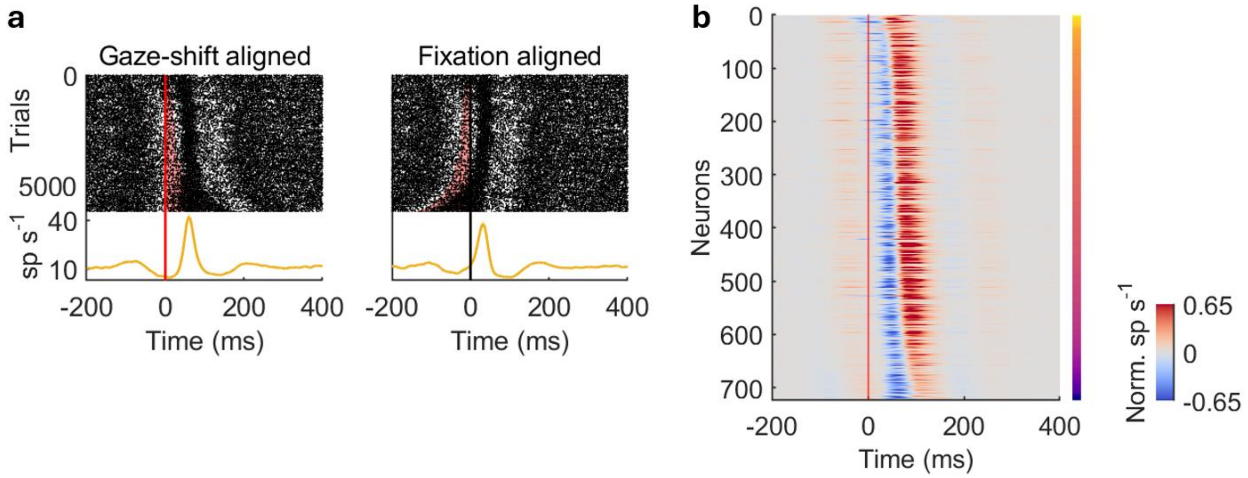

Figure S3. Example early-latency neuron and model-obtained PSTHs aligned with gaze-shift onset in the V1 population.

a) Raster plots and PSTHs aligned with gaze-shift onsets (left) and fixation onsets (right) for an example neuron with early latency. The enhancement locks in with gaze-shift onset instead of fixation onset in raster plots, and the enhancement amplitude is larger in the gaze-shift aligned PSTH than the fixation aligned PSTH.

b) Normalized PSTHs aligned with gaze-shift onset obtained from the two-stage model. The neurons are sorted by response latencies same as Fig. 1d. The two-stage model, which incorporates the actual sequence of gaze events, recapitulates not only the main response but also the weak suppression and enhancement components flanking the main response.
